# 

**Published:** 1885-03

**Authors:** 


CONTENTS.

Vol. 32 No. 3.

| | PAGE |
|---|---|
| Laughter | 1 |
| Strength is Not Health | 2 |
| Mental Exercises Necessary to Health | 2 |
| Food and Brain Work | 3 |
| A Cure for Styes | 3 |
| Explosive Mixtures | 4 |
| Rattle Snake Bite | 4 |
| Need of Rest | 6 |
| A Dead Tooth | 7 |
| Lecture on Physical Exercise | 7 |
| New Remedy for Asthma | 9 |
| Secret Remedies | 10 |
| What You Should Eat | 10 |
| Laughter as a Medicine | 11 |
| Acid Phosphate | 12 |
| How to Boil Water | 12 |
| Energy of a Sheet of Paper | 13 |
| Appetite | 13 |
| Premature Deaths | 14 |
| How to Remove Scars | 15 |
| Dietetic Treatment of Dyspepsia | 15 |
| Rice | 16 |
| Tobacco Smoke | 17 |
| A Trade as a Refuge | 19 |
| Danger in the Water Trough | 20 |
| A Good Disinfectant | 20 |
| Vaccination Against Yellow Fever | 20 |

